# Influenza-Associated Medical Visits Prevented by Influenza Vaccination in Young Children in Thailand, 2012–2014

**DOI:** 10.1093/jpids/piaa076

**Published:** 2021-04-03

**Authors:** Melissa A. Rolfes, Sonja J. Olsen, Wanitchaya Kittikraisak, Piyarat Suntarattiwong, Chonticha Klungthong, Damon Ellison, Joshua A. Mott, Tawee Chotpitayasunondh

**Affiliations:** 1 Influenza Division, Centers for Disease Control and Prevention, Atlanta, Georgia, USA;; 2 Influenza Program, Thailand Ministry of Public Health–US Centers for Disease Control and Prevention Collaboration, Nonthaburi, Thailand;; 3 Queen Sirikit National Institute of Child Health, Ministry of Public Health, Bangkok, Thailand;; 4 US Armed Forces Research Institute of Medical Sciences, Bangkok, Thailand

**Keywords:** burden, children, influenza, Thailand, vaccine effectiveness

## Abstract

Despite recommendations, few children aged 6–35 months in Thailand receive seasonal influenza vaccination. Using previously estimated incidence and vaccine effectiveness data from the period 2012–2014, we estimate that up to 121 000 medical visits could be prevented each year with 50% coverage and expanded recommendations to children aged <5 years.

Since 2009, Thailand has recommended influenza vaccination for children aged 6–35 months; however, uptake among children has been low, with 1% coverage nationally [1]. Given the burden of influenza in Thailand, the population-level impact of greater influenza vaccination of children could be substantial. Using data on vaccine effectiveness and incidence of influenza from studies at a large children’s hospital in Bangkok, we estimated the number of influenza virus infections, nationally, that could be prevented by increasing coverage among children aged 6–36 months. We further explored the benefits of expanding the vaccination recommendations to all children aged 6–59 months.

## METHODS

We estimated the number of medical visits for symptomatic influenza virus infections that could have been prevented by vaccination as the difference between the number of medical visits that occurred without any influenza vaccination and the number of illnesses that would have occurred with a given vaccine coverage and effectiveness.

To estimate the number of influenza-associated medical visits that occurred without any influenza vaccination among young children in Thailand, we calculated the annual incidence of influenza-associated medical visits among unvaccinated children in a prospective cohort conducted in Bangkok, Thailand from 2011 to 2015 [2]. The cohort consisted of children, aged <36 months at enrollment in 2011, who regularly obtained healthcare from the Queen Sirikit National Institute of Child Health (QSNICH) in Bangkok. Caregivers of enrolled children were contacted weekly to identify acute respiratory illness (ARI; defined as ≥2 of the following: fever, cough, sore throat, or runny nose) in the children. Children with ARI were brought to QSNICH for clinical assessment and testing for influenza using reverse-transcription polymerase chain reaction. Caregivers were contacted 1–2 weeks following onset to determine outcomes of illness. We calculated the incidence of influenza-associated medical visits from June to May for 2012, 2013, and 2014 and extrapolated rates to children aged 6–59 months in Thailand, using Thai census projections [3].

To estimate the number of influenza-associated medical visits that would have occurred with influenza vaccination, we multiplied the incidence rate among the unvaccinated by the population susceptible to influenza virus infection. The proportion of the population susceptible was estimated by 1 – (vaccine effectiveness × vaccine coverage). We used previously published estimates of vaccine effectiveness, which were estimated from the same pediatric population in Bangkok (Supplementary Table 1) [4, 5]. We assumed 1% annual influenza vaccination coverage, which was the national coverage estimated in 2011 [1], but also explored higher coverage of 20% and 50%. To estimate the number of influenza-associated medical visits prevented by vaccination, we subtracted the estimated medical visits under each vaccination coverage level from the estimated number of medical visits without vaccination.

To estimate uncertainty intervals around estimates, we used 5000 Monte Carlo simulations, assuming a Poisson distribution for incidence and a log-normal distribution for vaccine effectiveness (VE) (truncated at 0, assuming vaccination does not increase the risk of influenza). For each simulation, we drew a random value from each assumed distribution and used the value to estimate the number of prevented medical visits. We report the median and 95% uncertainty intervals for the simulations. We stratified all analyses and simulations by year, age group (6–35 months and 36–59 months), and outpatient visits or hospitalizations. We conducted analyses using R version 3.5.3 software [6].

## RESULTS

Among unvaccinated children aged 6–35 months in the prospective cohort, the annual incidence of influenza-associated medical visits varied from 48 to 113 visits per 1000 during 2012–2014. Applying the incidence estimates to the Thai pediatric population aged 6–35 months, we estimate that 83 000–205 000 outpatient visits and 10 000–25 000 hospitalizations due to influenza would have occurred each year without any vaccination (Supplementary Table 2). With an estimated VE that varied between 26% and 64% from 2012 through 2014, and coverage of 1%, we estimated that 190–1300 outpatient visits and 1–146 hospitalizations were prevented by vaccination, depending on the year (Supplementary Table 3). Influenza vaccine coverage of 20% would have prevented 4400–28 000 total outpatient or inpatient medical visits and 50% coverage would have prevented 11 000–69 000 total medical visits among children aged 6–35 months.

**Figure 1.**
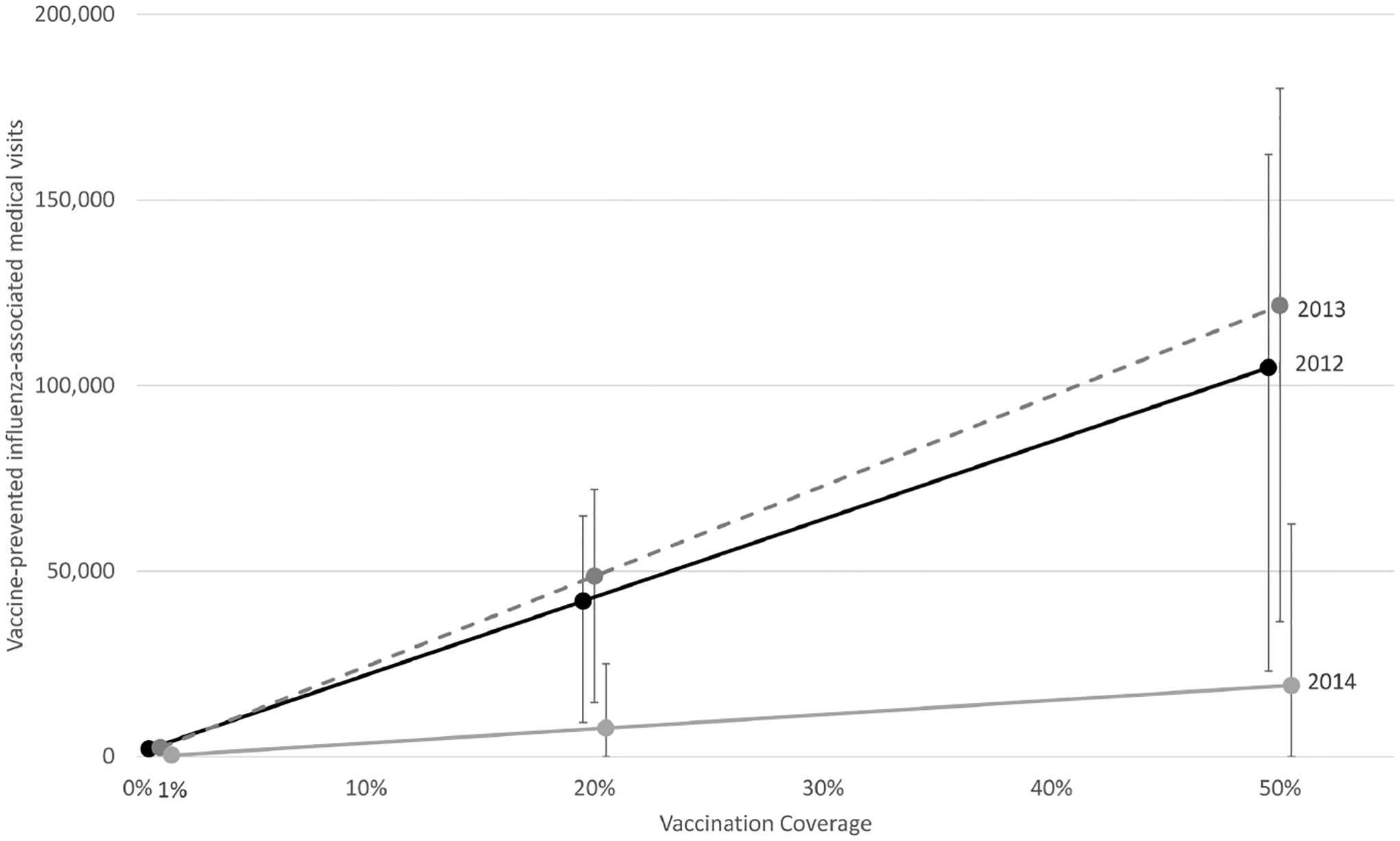
Estimated influenza-associated outpatient and inpatient medical visits prevented by varying influenza vaccination coverage levels among children aged 6–59 months in Thailand during 2012–2014. Error bars represent 95% uncertainty intervals from 5000 Monte Carlo simulations.

An additional 64 000–173 000 total medical visits occurred among children aged 36–59 months, for an overall estimate of 161 000–387 000 medical visits due to influenza each year in children <5 years old in Thailand. If the recommendations for influenza vaccination were expanded to include all children aged 6–59 months and 20% coverage were achieved, then 7700–49 000 total medical visits would have been prevented per year. Increasing coverage to 50% would have prevented between 19 000 and 121 000 total medical visits, depending on the year (Figure 1 and Supplementary Table 3).

## DISCUSSION

Among children aged 6–59 months in Thailand, we estimate that without influenza vaccination, influenza resulted in as few as 161 000–387 000 medical visits each year during 2012–2014, affecting approximately 11% of this pediatric population. When the influenza vaccine is well-matched to the circulating influenza virus subtypes, as it was in 2012 and 2013 in Thailand, vaccination can substantially reduce the burden and cost associated with influenza [2].

Currently in Thailand, influenza vaccination is recommended for children aged 6–35 months. However, coverage is low, with 1% of children being vaccinated in 2011 [1]. If national coverage remained at 1% from 2012–2014, we estimated that influenza vaccination prevented <2000 medical visits each season. However, by improving influenza vaccination coverage to 50% in children aged 6–35 months, as many as 69 000 influenza-associated medical visits would have been prevented in 2013, including prevention of 4900 hospitalizations.

Influenza vaccination is commonly recommended for children younger than 36 months to prevent severe complications of influenza, including pneumonia and hospitalization. However, the incidence of medically attended influenza in Bangkok was similar between children aged 6–36 months and those aged 36–59 months, an age group that is not currently included in the Thai influenza vaccination recommendations. In our analysis, we found that expanding the recommended age for influenza vaccination to all children aged 6–59 months, and achieving at least 20% coverage, would prevent thousands more influenza-associated medical visits, primarily in the outpatient setting. In 2013, when the incidence of influenza was greatest in the cohort in Bangkok and the VE was 64%, vaccinating 50% of all children aged 6–59 months could have prevented 45 000 medical visits.

The greatest population benefit of influenza vaccination occurs when the influenza vaccine is antigenically well-matched to circulating influenza viruses. However, even in seasons where one vaccine component is mismatched, there can be population benefits of influenza vaccination given the typically large burden of influenza and due to reduced incidence of other well-matched influenza types and subtypes. For example, the influenza A(H3N2) viruses that predominated in Thailand, and most of the world, in 2014 were antigenically distinct from the A(H3N2) viruses contained in the 2014 Southern Hemisphere vaccine. The estimated VE against medically attended illness associated with any influenza in the Thai children was 26% and not statistically significant [5]. While the uncertainty bounds included 0, we estimated some prevented medical visits in 2014, perhaps reflecting effectiveness of the vaccine against influenza (H1N1)pdm09 and B viruses that also circulated in September–December 2014 and were well-matched to the vaccine [5].

There are several limitations to our analysis. We assumed that the incidence we observed was generalizable; however, because we encouraged cohort participants to seek care when they were ill, the estimated incidence of medically attended respiratory illness may be higher than in the general pediatric population. Additionally, our inpatient estimates may not be generalizable because there were few hospitalizations in our cohort, particularly in children aged 36–59 months. However, several other studies from Thailand have reported influenza incidence estimates similar to the incidence estimates observed in the Bangkok cohort, including an estimated overall incidence of influenza of 59.4 per 1000 across all ages [7] and influenza-associated pneumonia among hospitalized children aged <5 years in rural Thailand of 2.4 per 1000 [8]. Prior estimates of influenza-associated hospitalized pneumonia were lower than the rate of hospitalization estimated from our cohort, but that would be expected given that pneumonia is one of many complications that of influenza could result in hospitalization [9, 10]. Finally, our method estimated the direct benefit of influenza vaccination to the child, which did not account for the potential indirect benefit to families and other contacts of the child and may overestimate the reduced burden compared with other methods [11].

Annual epidemics of influenza result in thousands of medical visits and hospitalizations among young children in Thailand; and influenza vaccination is an important means to prevent illness and reduce the burden to the healthcare system. However, during the study period, influenza vaccination was rare among children in Thailand. We estimate that increasing vaccine coverage will result in a far greater reduction in influenza-associated illness and healthcare utilization. Some strategies to increase coverage include educating healthcare providers and the public about the benefits and effectiveness of influenza vaccination, encouraging providers to make a recommendation for influenza vaccination to parents [12, 13], targeting communications to parents of young children during vaccine campaigns, and making vaccination more accessible by purchasing more doses or locally producing influenza vaccines [14].

## Supplementary Material

Supplementary data
