# Supplementary material for: Influenza-Associated Medical Visits Prevented by Influenza Vaccination in Young Children in Thailand, 2012–2014
Source: J Pediatric Infect Dis Soc. Author manuscript; Available in PMC 2024 Jan 10. (PMC10776449; doi:10.1093/jpids/piaa076)
Supplement: Supplementary data [file NIHMS1956094-supplement-Supplementary_data.docx]

**Influenza-associated Medical Visits Prevented by Influenza Vaccination in Young Children in Thailand, 2012–2014**

Melissa A Rolfes^1^, Sonja J. Olsen^1^, Wanitchaya Kittikraisak^2^, Piyarat Suntarattiwong^3^, Chonticha Klungthong^4^, Damon Ellison^4^, Joshua A. Mott^2^, Tawee Chotpitayasunondh^3^

^1^Influenza Division, U.S. Centers for Disease Control and Prevention, Atlanta, Georgia, USA; ^2^ Influenza Program, Thailand MOPH – U.S. CDC Collaboration, Nonthaburi, Thailand; ^3^Queen Sirikit National Institute of Child Health, Ministry of Public Health, Bangkok, Thailand; ^4^U.S. Armed Forces Research Institute of Medical Sciences, Bangkok, Thailand

**Supplemental table 1:** Predominant circulating influenza viruses and effectiveness of influenza vaccination among young children in Thailand, 2012-2014

| Year | Predominant influenza virus in circulation | Vaccine effectiveness  (95% CI) * | Vaccine composition [1-3] |
| --- | --- | --- | --- |
| 2012 | Mixed: B and A(H1N1) | 64% (12%, 85%) | A(H1N1): A/California/7/2009-like  A(H3N2): A/Perth/16/2009-like  B: B/Brisbane/60/2008-like (Victoria lineage) |
| 2013 | A(H3N2) | 64% (21%, 84%) | A(H1N1): A/California/7/2009-like  A(H3N2): A/Victoria/361/2011-like  B: B/Wisconsin/1/2010-like (Yamagata lineage) |
| 2014 | Mixed: B (Yamagata) and A(H3N2) | 26% (-47%, 63%) | A(H1N1): A/California/7/2009-like  A(H3N2): A/Texas/50/2012-like ^†^  B: B/Massachusetts/2/2012-like (Yamagata lineage) |

*95% confidence intervals (CI). In 2012, vaccine effectiveness against medically attended influenza, of any type as either an outpatient or admitted to the hospital, was estimated among children aged 6–36 months who were fully vaccinated with influenza vaccine [4]. In 2013 and 2014, vaccine effectiveness against medically attended influenza, of any type as either an outpatient or admitted to the hospital, was estimated among children aged 7–60 months who were fully vaccinated with influenza vaccine [5].

^†^ In 2014, the A/Texas/50/2012-like H3N2 virus was antigenically like the cell-propagated prototype A/Victoria/362/2011 virus included in the 2013 Southern Hemisphere influenza vaccines [3].

**Supplemental table 2:** Incidence of medically-attended influenza and number of influenza-associated medical visits that occurred without vaccination by year and severity of illness for children aged 6–59 months, Thailand, 2012–2014

|  | Outpatient medical visits | | Hospitalizations | | Overall | |
| --- | --- | --- | --- | --- | --- | --- |
| Year | Incidence in the unvaccinated (per 1,000 child-years, 95% UI*) | Estimated burden without vaccination (95% UI) | Incidence in the unvaccinated (per 1,000 child-years, 95% UI) | Estimated burden without vaccination (95% UI) | Incidence in the unvaccinated (per 1,000 child-years, 95% UI) | Estimated burden without vaccination (95% UI) |
| Aged 6-35 months | |  |  |  |  |  |
| 2012 | 78.2 (53.0, 106.0) | 155,587 (105,397, 210,795) | 12.6 (2.5, 25.2) | 25,095 (5,019, 50,189) | 90.8 (63.1, 121.1) | 180,681 (125,473, 240,908) |
| 2013 | 104.4 (75.5, 135.5) | 204,568 (147,985, 265,503) | 8.9 (2.2, 17.8) | 17,410 (4,353, 34,820) | 113.3 (82.2, 146.6) | 221,978 (161,043, 287,266) |
| 2014 | 42.6 (16.0, 74.6) | 83,026 (31,135, 145,296) | 5.3 (0.0, 16.0) | 10,378 (0, 31,135) | 47.9 (21.3, 79.9) | 93,404 (41,513, 155,674) |
| Aged 36-59 months | |  |  |  |  |  |
| 2012 | 81.3 (27.1, 149.1) | 129,398 (43,133, 237,230) | 27.1 (0.0, 67.8) | 43,133 (0, 107,832) | 108.4 (40.7, 189.7) | 172,531 (64,699, 301,930) |
| 2013 | 95.8 (43.6, 156.8) | 150,206 (68,275, 245,791) | 0.0 (0.0, 0.0) | 0 (0, 0) | 95.8 (43.6, 156.8) | 150,206 (68,275, 245,791) |
| 2014 | 41.2 (0.0, 96.0) | 64,132 (0, 149,641) | 0.0 (0.0, 0.0) | 0 (0, 0) | 41.2 (0.0, 96.0) | 64,132 (0, 149,641) |
| Overall |  |  |  |  |  |  |
| 2012 | 78.7 (55.3, 104.2) | 281,796 (198,019, 373,190) | 14.9 (4.3, 27.6) | 53,313 (15,232, 99,010) | 93.6 (68.1, 121.2) | 335,109 (243,716, 434,119) |
| 2013 | 102.7 (77.9, 129.2) | 362,074 (274,677, 455,870) | 7.1 (1.8, 14.2) | 24,971 (6,243, 49,941) | 109.7 (83.2, 138.1) | 387,045 (293,405, 486,927) |
| 2014 | 42.2 (19.2, 69.1) | 148,006 (67,276, 242,192) | 3.8 (0.0, 11.5) | 13,455 (0, 40,365) | 46.0 (23.0, 72.9) | 161,461 (80,731, 255,647) |

* 95% uncertainty intervals (UI) estimated from 5,000 Monte Carlo simulations.

**Supplemental table 3:** Estimated influenza-associated outpatient medical visits and hospitalizations prevented by influenza vaccination among children aged 6–59 months, Thailand, 2012–2014, by year with varying levels of influenza vaccine coverage

| Age group | Year | Vaccine coverage | Prevented outpatient visits (95% UI*) | Prevented hospitalizations (95% UI) | Prevented medical visits  (outpatient or hospitalizations;  95% UI) |
| --- | --- | --- | --- | --- | --- |
| 6-35 months | 2012 | 1% | 961 (207, 1,568) | 146 (16, 341) | 1,117 (238, 1,773) |
|  |  | 20% | 19,227 (4,137, 31,352) | 2,921 (316, 6,816) | 22,343 (4,759, 35,466) |
|  |  | 50% | 48,067 (10,343, 78,380) | 7,302 (791, 17,040) | 55,857 (11,897, 88,664) |
|  | 2013 | 1% | 1,285 (405, 1,956) | 98 (4, 244) | 1,382 (444, 2,112) |
|  |  | 20% | 25,695 (8,101, 39,114) | 1,957 (75, 4,890) | 27,639 (8,889, 42,245) |
|  |  | 50% | 64,238 (20,252, 97,784) | 4,892 (189, 12,224) | 69,098 (22,222, 105,612) |
|  | 2014 | 1% | 190 (0, 662) | 1 (0, 136) | 218 (0, 740) |
|  |  | 20% | 3,805 (0, 13,244) | 23 (0, 2,720) | 4,351 (0, 14,804) |
|  |  | 50% | 9,513 (0, 33,110) | 59 (0, 6,799) | 10,879 (0, 37,011) |
| 36-59 months | 2012 | 1% | 760 (100, 1,717) | 233 (0, 768) | 1,010 (169, 2,136) |
|  |  | 20% | 15,199 (1,996, 34,340) | 4,654 (0, 15,359) | 20,198 (3,387, 42,721) |
|  |  | 50% | 37,997 (4,990, 85,851) | 11,635 (0, 38,399) | 50,495 (8,468, 106,803) |
|  | 2013 | 1% | 897 (233, 1,689) | -- | 902 (239, 1,719) |
|  |  | 20% | 17,936 (4,669, 33,771) | -- | 18,042 (4,776, 34,377) |
|  |  | 50% | 44,839 (11,672, 84,428) | -- | 45,105 (11,941, 85,941) |
|  | 2014 | 1% | 122 (0, 631) | -- | 118 (0, 615) |
|  |  | 20% | 2,444 (0, 12,622) | -- | 2,365 (0, 12,292) |
|  |  | 50% | 6,110 (0, 31,554) | -- | 5,913 (0, 30,730) |
| All children, aged 6-59 months | 2012 | 1% | 1,743 (370, 2,791) | 305 (50, 670) | 2,096 (461, 3,247) |
|  |  | 20% | 34,870 (7,402, 55,810) | 6,096 (1,008, 13,409) | 41,920 (9,220, 64,938) |
|  |  | 50% | 87,174 (18,504, 139,525) | 15,239 (2,521, 33,522) | 104,800 (23,051, 162,344) |
|  | 2013 | 1% | 2,267 (721, 3,395) | 141 (13, 355) | 2,430 (728, 3,600) |
|  |  | 20% | 45,344 (14,418, 67,890) | 2,817 (261, 7,095) | 48,599 (14,568, 72,001) |
|  |  | 50% | 113,360 (36,046, 169,726) | 7,042 (652, 17,737) | 121,498 (36,421, 180,003) |
|  | 2014 | 1% | 354 (0, 1,125) | 7 (0, 182) | 384 (0, 1,253) |
|  |  | 20% | 7,081 (0, 22,497) | 146 (0, 3,644) | 7,673 (0, 25,069) |
|  |  | 50% | 17,703 (0, 56,243) | 366 (0, 9,110) | 19,183 (0, 62,673) |

* 95% uncertainty intervals (UI) estimated from 5,000 Monte Carlo simulations.

**Disclaimers**

The findings and conclusions in the report are those of the authors and do not necessarily represent the official position of the Centers for Disease Control and Prevention. Additionally, material has been reviewed by the Walter Reed Army Institute of Research. There is no objection to its presentation and/or publication. The opinions or assertions contained herein are the private views of the authors, and are not to be construed as official, or as reflecting true views of the Department of the Army or the Department of Defense.

**References**

1. World Health Organization. Recommended composition of influenza virus vaccines for use in the 2012 southern hemisphere influenza season. Available at: <https://www.who.int/influenza/vaccines/virus/recommendations/2012south/en/>. Accessed January 9, 2020.

2. World Health Organization. Recommended composition of influenza virus vaccines for use in the 2013 southern hemisphere influenza season. Available at: <https://www.who.int/influenza/vaccines/virus/recommendations/2013_south/en/>. Accessed January 9, 2020.

3. World Health Organization. Recommended composition of influenza virus vaccines for use in the 2014 southern hemisphere influenza season. Available at: <https://www.who.int/influenza/vaccines/virus/recommendations/2014_south/en/>. Accessed January 9, 2020.

4. Kittikraisak W, Suntarattiwong P, Levy J, et al. Influenza vaccination coverage and effectiveness in young children in Thailand, 2011-2013. Influenza Other Respir Viruses **2015**.

5. Kittikraisak W, Suntarattiwong P, Ditsungnoen D, et al. Effectiveness of the 2013 and 2014 Southern Hemisphere Influenza Vaccines Against Laboratory-confirmed Influenza in Young Children Using a Test-negative Design, Bangkok, Thailand. Pediatr Infect Dis J **2016**; 35(10): e318-25.
